# A novel culture method that sustains ERα signaling in human breast cancer tissue microstructures

**DOI:** 10.1186/s13046-020-01653-4

**Published:** 2020-08-17

**Authors:** Ana Luísa Cartaxo, Marta F. Estrada, Giacomo Domenici, Ruben Roque, Fernanda Silva, Emilio J. Gualda, Pablo Loza-Alvarez, George Sflomos, Cathrin Brisken, Paula M. Alves, Saudade André, Catarina Brito

**Affiliations:** 1 iBET, Instituto de Biologia Experimental e Tecnológica, Oeiras, Portugal; 2 Instituto de Tecnologia Química e Biológica António Xavier, Oeiras, Portugal; 3 IPOLFG, Instituto Português de Oncologia de Lisboa Francisco Gentil, Lisbon, Portugal; 4 CEDOC, Chronic Diseases Research Centre, NOVA Medical School, Universidade NOVA de Lisboa, Lisbon, Portugal; 5 ICFO, Institut de Ciències Fotòniques, The Barcelona Institute of Science and Technology, Castelldefels, Barcelona, Spain; 6 Swiss Institute for Experimental Cancer Research, School of Life Sciences, Ecole polytechnique fédérale de Lausanne (EPFL), Lausanne, Switzerland

**Keywords:** Cancer, Patient-derived cancer models, 17-β-estradiol, Estrogen receptor alpha, Fulvestrant, Encapsulation, Alginate

## Abstract

**Background:**

Estrogen receptor α (ERα) signaling is a defining and driving event in most breast cancers; ERα is detected in malignant epithelial cells of 75% of all breast cancers (classified as ER-positive breast cancer) and, in these cases, ERα targeting is the main therapeutic strategy. However, the biological determinants of ERα heterogeneity and the mechanisms underlying therapeutic resistance are still elusive, hampered by the challenges in developing experimental models recapitulative of intra-tumoral heterogeneity and in which ERα signaling is sustained. Ex vivo cultures of human breast cancer tissue have been proposed to retain the original tissue architecture, epithelial and stromal cell components and ERα. However, loss of cellularity, viability and ERα expression are well-known culture-related phenomena.

**Methods:**

BC samples were collected and brought to the laboratory. Then they were minced, enzymatically digested, entrapped in alginate and cultured for 1 month. The histological architecture, cellular composition and cell proliferation of tissue microstructures were assessed by immunohistochemistry. Cell viability was assessed by measurement of cell metabolic activity and histological evaluation. The presence of ERα was accessed by immunohistochemistry and RT-qPCR and its functionality evaluated by challenge with 17-β-estradiol and fulvestrant.

**Results:**

We describe a strategy based on entrapment of breast cancer tissue microstructures in alginate capsules and their long-term culture under agitation, successfully applied to tissue obtained from 63 breast cancer patients. After 1 month in culture, the architectural features of the encapsulated tissue microstructures were similar to the original patient tumors: epithelial, stromal and endothelial compartments were maintained, with an average of 97% of cell viability compared to day 0. In ERα-positive cases, fibers of collagen, the main extracellular matrix component in vivo, were preserved. ERα expression was at least partially retained at gene and protein levels and response to ERα stimulation and inhibition was observed at the level of downstream targets, demonstrating active ER signaling.

**Conclusions:**

The proposed model system is a new methodology to study ex vivo breast cancer biology, in particular ERα signaling. It is suitable for interrogating the long-term effects of anti-endocrine drugs in a set-up that closely resembles the original tumor microenvironment, with potential application in pre- and co-clinical assays of ERα-positive breast cancer.

## Background

Breast cancer (BC) is the most commonly diagnosed cancer among women worldwide [1]. It is a heterogeneous disease with distinct biological features and clinical outcomes. Almost 75% of diagnosed BC express estrogen receptor-alpha (ERα), being classified as ERα-positive (ER+) BC [2]. ERα acts as a ligand-dependent transcription factor for genes associated with cell survival, proliferation, and tumor growth [3]. Therefore, targeting the ERα-signaling pathway is the main therapeutic strategy for the treatment of ER+ BCs. Nonetheless, the disease often progresses in 30% of the patients undergoing hormonal therapy due to resistance [2]. Thus, there is a need to select patients that would respond to endocrine therapy and to elucidate the molecular mechanisms behind endocrine resistance, as well as to identify biomarkers that predict drug response and resistance and novel therapeutic targets in resistant tumors.

When cultured in classical 2D monolayers, ER+ BC cell lines fail in recapitulating the typical intratumoral ERα heterogeneity [4] and, due to cell confluency, cannot be kept continuously for more than 1 week [5], hampering the possibility to perform cycles of drug treatment for more than 1 week. Only a few ER+ cell lines can generate xenografts in mice, requiring supplementation with estrogen [6]. Recently, an estrogen supplementation-independent in vivo model was reported, based on intraductal implantation of ER+ tumor cells. The demonstration that the intraductal but not the mammary fat pad microenvironment favors epithelial malignant cells of the luminal subtype, consolidated the role of the tumor microenvironment (TME) in sustaining ER+ tumor cells [6]. Although there is a report showing that it is possible to propagate normal primary breast ER+ cells in 2D [7], there are no reports for propagation of primary ER+ BC cells using this culture system. In fact, ER+ BC primary cells cultured in 2D loose cellularity and ERα expression after a short culture period.

Ex vivo cultures have been explored to sustain ER+ malignant epithelial cells within the original BC microenvironment [8]. Typically, these models retain tissue architecture and heterogeneity for short periods of time, around 3 to 4 days of culture [7, 9]. Naipal et al. reported extension of culture time up to 7 days by exploring dynamic culture conditions [10]. Recently, Muraro et al. reported high cell viability and maintenance of ER expression up to 14 days in culture, when combining a collagen scaffold and a medium perfusion system [8]. Nonetheless, this methodology supports BC tissue maintenance by taking advantage of collagen scaffolds, biologically active animal-derived biomaterials which bring variability, as well as environmental and ethical concerns [11].

Here, we hypothesized that retention of the original microenvironment would favor the maintenance of ER+ BC phenotype and ERα signaling. We implemented an ex vivo strategy based on the encapsulation of tissue microstructures in alginate, an inert biomaterial, combined with dynamic culture, aiming to maintain the original tissue structure, cell populations and extracellular matrix (ECM). We have recently shown that alginate microencapsulation of cancer cell spheroids and TME cellular components promotes tumor-stromal crosstalk and retention of secreted ECM components towards reconstruction of TME features [12, 13]. Therefore, we reasoned that by using alginate encapsulation to promote the original TME retention, while resourcing to dynamic culture to guarantee efficient diffusion of nutrients and oxygen, tissue microstructures would retain architectural integrity and potentially ER signaling.

## Methods

### Ethics statement

BC samples were collected at the Lisbon Oncology Hospital (Instituto Português de Oncologia de Lisboa Francisco Gentil – IPOLFG). The use of patient material was approved by the IPOFLG ethics committee and all patients have signed an informed consent form to agree to donate the material for research purposes. All tissues were anonymized before transfer to the laboratory for further processing.

### Cell culture

MDA-MB-231 cell line was obtained from the American Type Culture Collection (ATCC). MDA-MB-231 cells were cultured in Dulbecco’s Modified Eagle Medium (DMEM) high glucose and pyruvate medium (Gibco), supplemented with 10% (v/v) fetal bovine serum (FBS, Gibco) and 1% (v/v) Penicillin/Streptomycin (P/S, Gibco) at 37 °C in 5% CO_2_. Mycoplasma contamination was routinely checked.

### Collection and processing of patient material

**Fig. 1:**
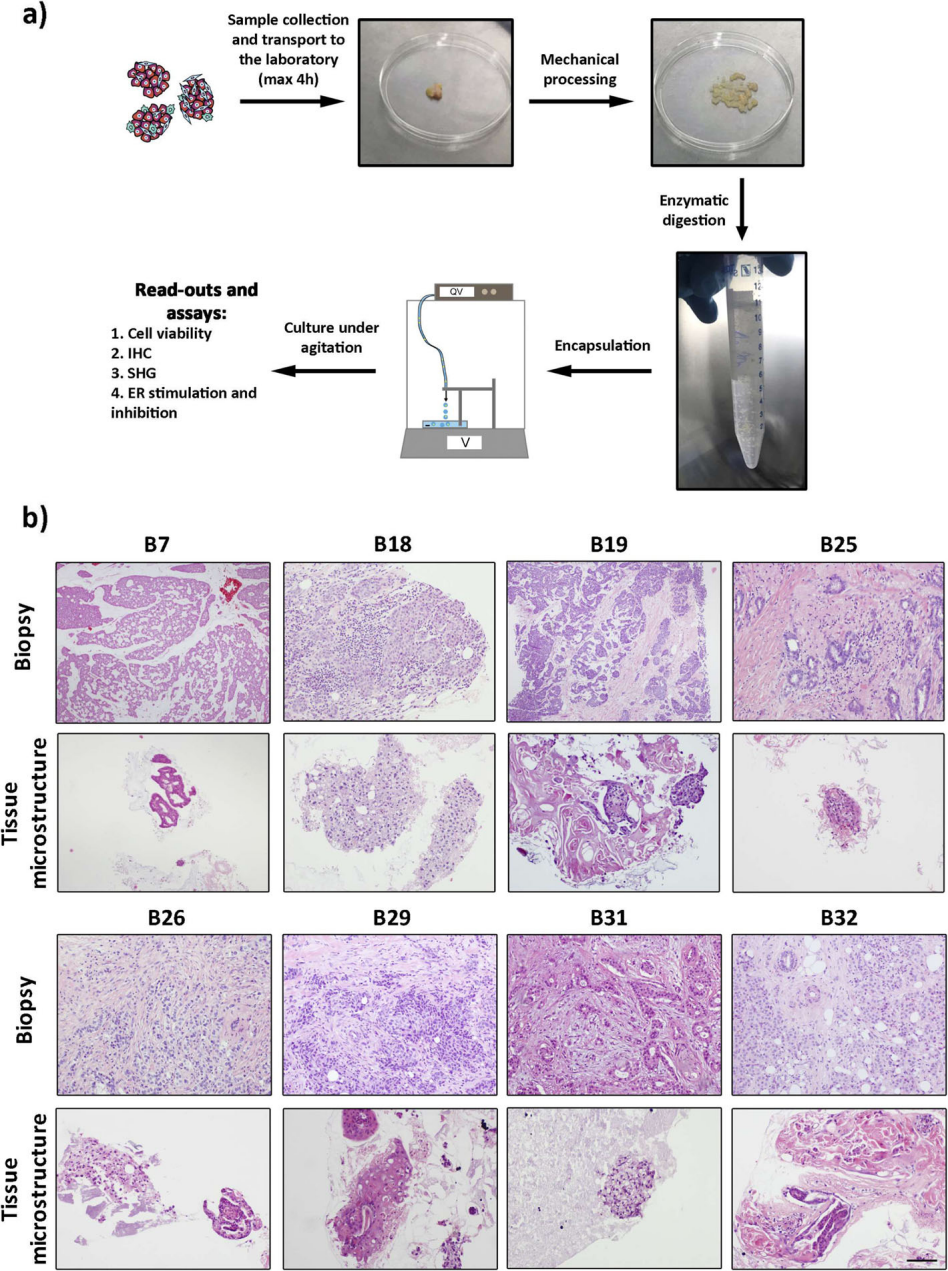
Alginate encapsulated tissue microstructures maintained parental tumor architecture. **a** Experimental workflow for the establishment of long-term cultures of BC patient-derived tissue microstructures: samples were collected at the hospital and brought to the laboratory within 1–3 h of surgery. Tissue samples were mechanical processed and subjected to mild enzymatic digestion. The obtained BC tissue microstructures were encapsulated in alginate and cultured for up to 1 month. Along culture, tissue microstructures were interrogated: cell viability assessment, immunohistochemistry analysis (IHC), Second Harmonic Generation (SHG) microscopy and estrogen receptor α (ER) stimulation and inhibition were performed. **b** Hematoxylin and eosin of biopsy (top row) and corresponding encapsulated microstructures at 1 month of culture (bottom row) (scale: 200 μm)

This study was elaborated on treatment-naïve patient-derived BC tissue. The method for processing and culture was successfully applied to 63 female breast tumors (Table 1). Tumor samples were collected during surgery and immediately submerged in phenol red-free DMEM/F-12 (Gibco), supplemented with 1% (v/v) P/S (Gibco) and 10% (v/v) FBS (Gibco). Samples were kept at 4 °C and transported to the laboratory within 1 to 3 h after surgery (Fig. 1a). Sixty-three BC samples were collected, with an average weight of 315 ± 225 mg (Figure S1).

**Table 1:** Clinico-pathological parameters of the breast cancer patients. pT: primary tumor; pN: primary node

Clinico-pathological parameters	(n)	
Female tumor samples	63	
Mean age at diagnosis	62 (42–89)	
**Hormone receptors**	**(n)**	**Percentage of tumors**
ERα status	59	94
PR status	51	81
HER2 status	11	17
Triple negative status	1	2
**Histological subtype**	**(n)**	**Percentage of tumors**
Invasive breast carcinoma of no-special type (NST)	51	81
Lobular	10	16
Mucinous	2	3
**Tumor grade**	**(n)**	**Percentage of tumors**
1	2	3
2	50	79
3	9	14
Not defined	2	3
**Tumor size**	**(n)**	**Percentage of tumors**
pT1	38	60
pT2	22	35
pT3	3	5
**Lymph node involvement status**	**(n)**	**Percentage of tumors**
pN0	41	65
pN1	21	33
pN2	1	2

Tissue samples were mechanically dissociated with two surgical scalpels to obtain pieces of 1 to 2 mm of diameter. Subsequently, the minced tissue was resuspended in phenol red-free DMEM/F-12, HEPES medium (Gibco) containing 0.09 U/mL of Collagenase A (Roche), 30 U/mL of Benzonase (Merck Millipore), 10% (v/v) FBS (Gibco) and 1% (v/v) P/S (Gibco). Digestion was performed in an incubator at 37 °C, in a humidified atmosphere containing 5% CO_2_. After 12–15 h of enzymatic digestion, tumor fragments (tissue microstructures, average of 1 mm^3^) were sedimented by centrifugation at 100x g for 5 min at 4 °C and washed with Phosphate-Buffered Saline (PBS; Life Technologies) (Fig. 1a).

### Tissue microstructure encapsulation and culture

Tissue microstructures were entrapped in alginate, employing protocols previously developed by our team [13]. Briefly, tissue microstructures were dispersed in 1 mL of 2% (w/v) of Ultrapure Ca^2+^ MVG alginate (UP MVG NovaMatrix, Pronova Biomedical, Oslo, Norway) dissolved in NaCl 0.9% (w/v). Encapsulation was performed using an electrostatic bead generator (Nisco VarV1, Zurich, Switzerland), with an air flow rate of 10 mL/h, at 5.3 V under air pressure of 1 bar, using a 1.1 mm nozzle. The resulting alginate droplets containing tissue micro fragments (1–2 fragments/droplet) were cross-linked in a 100 mM CaCl_2_/10 mM HEPES (pH 7.4) solution for 10 min, washed three times in a 0.9% (w/v) NaCl solution and finally equilibrated in culture medium. Encapsulated tissue microstructures were then transferred into 6-well plates and placed under orbital shaking (100 rpm), in a humidified incubator, with 5% CO_2_. Encapsulated tissue microstructures cultures were maintained up to 30 days, with 50% medium exchange every 3–4 days (Fig. 1a). Cultures were maintained in human mammary epithelial cell (HMEC) culture medium: DMEM/F12 phenol red free with 1% P/S (v/v) solution (both from Life Technologies), 5 ng/mL Epidermal Growth Factor (EGF), 10 μg/mL Insulin, 0.5 μg/mL Hydrocortisone, 0.5 μg/mL Transferrin, 0.1 mM Isoprotenol, 0.1 mM Ethanolamine, 0.1 mM O-Phosphoethanolamine, 70 μg/mL Bovine Pituitary Extract (all reagents are from Sigma-Aldrich) and 100 μg/mL Primocin (InvivoGen Europe). Non-encapsulated tissue microstructures were maintained under the same culture conditions. Encapsulated tissue microstructures were assessed for cell viability, architecture, cell populations, ECM deposition, ERα presence and signaling, as described below; the extent of assessment performed for each sample was determined by the initial sample size.

### Cell viability assessment

Cell viability was correlated with resazurin reduction capacity (PrestoBlue™ Cell Viability Reagent, ThermoFischer Scientific), according to manufacturer’s instructions. Encapsulated and non-encapsulated samples were incubated for 1 h with PrestoBlue reagent in culture medium, at 37 °C, in a humidified atmosphere incubator, containing 5% CO_2_. Medium was sampled in quadruplicate and resazurin reduction evaluated by fluorescence detection (ext/em 560/590 nm) in a fluorimeter (Infinite®200 PRO NanoQuant, Tecan Trading AG). Resazurin reduction was evaluated for 1 month, once a week. Data is represented as fold-change in resazurin reduction relative to the first week of the assay.

### Histological and immunohistochemistry analysis

Samples were collected after 1 month of culture and alginate capsules were de-polymerized with 50 mM EDTA for 5 min at RT. De-encapsulated tissue microstructures were centrifuged at 300x g, 5 min at 4 °C, washed with PBS, fixed with formol overnight at RT. For paraffin cell-block preparation, the cellular suspension was centrifuged for 5 min, at 1270x g, resuspended in 10% (v/v) buffered formalin (VWR BDH Chemicals, ref. 9713.9010) to which a drop of haematoxylin was added for specimen counterstain, and stored in a 1.5 mL microtube. The remaining supernatants were subjected to a second centrifugation, for 5 min, at 1990x g. The supernatant was discarded and four drops of liquefied HistoGel (Thermo Scientific, ref. HG-4000-012) were added to the pellet. After gentle homogenization with a Pasteur pipette and centrifugation for 2 min, at 1990x g, the sample was placed at − 20 °C for 5 min to solidify. The cone shape solidified sample was removed from the microtube, cut along the meridional section and placed in a biopsy cassette, which was then immerged in a container with buffered formalin to be included in paraffin. After processing, the samples were sectioned and stained with hematoxylin and eosin (H&E) (Dako CoverStainer for H&E equipment, Agilent, Santa Clara, CA, USA). Paraffin blocks were sectioned (3 μm) for H&E and immunohistochemical staining. Immunohistochemistry (IHC) was carried out using standard procedures implemented at IPOLFG; antigen retrieval was done using Cell Conditioning 1 (CC1, Ventana) and tissue staining was performed using an automated IHC/ISH slide staining Ventana BenchMark Ultra (all from Ventana Medical Systems, Inc). Antibodies and details on the protocol used are indicated in Table S1. Histologic analysis was performed by an expert breast pathologist. IHC analysis was performed for cultures derived from BC samples of 18 patients. Due to primary material limitations, E-cadherin, CD45, ki-67, ER and p63 levels were assessed in 8 different samples; vimentin was assessed in 9 and CD31 in 2.

### Multi-photon microscopy

Fibrillar Collagen was assessed by multi-photon microscopy. After 1 month in culture, encapsulated tissue microstructures were collected, fixed in PFA 4% (w/v) in PBS for 30 min, washed thrice with PBS and kept at 4 °C until further analysis. Samples were imaged with two-photon-excited fluorescence (TPEF), second harmonic generation (SHG) and infrared (IR) absorption in a home-made multiphoton microscope [14]. The excitation laser was a Ti:Sapphire at 810 nm and the laser power, at entrance of the microscope, was of 40 mW. Initial tests performed with 100 mW resulted in no observable sample damage. The Illumination objective was an Olympus 25 × 1.05 W. The TPEF signal was collected through a photomultiplier tube (PMT) in backward direction (using a LP410 filter) while IR absorption and SHG (405/25 filter) were collected in forward direction through a Nikon 25 × 1.10 W objective, using a photodetector and a PMT respectively. During acquisition, 3–4 images were averaged to reduce noise.

### Challenge with ERα agonist and antagonist

At day 28–30 of culture, encapsulated BC tissue microstructures were stimulated with 10 nM 17-β-estradiol (Sigma-Aldrich). Three days before 17-β-estradiol challenge, encapsulated tissue microstructures were washed thrice with PBS and were then kept in phenol red-free HMEC medium without insulin, hydrocortisone and EGF, which may trigger activation or phosphorylation of ER [15–20]. Alternatively, a 50% culture medium exchange was performed by the time of 17-β-estradiol challenge. Control wells were also included, in which only ethanol (17-β-estradiol vehicle) was added to a final concentration of 0.001% (v/v). After 24 h of exposure, encapsulated tissue microstructures were collected and alginate dissolved (as described in section 6 of Materials and Methods). Challenge with 17-β-estradiol was performed in encapsulated microstructures derived from 16 different patients, of which 9 in depleted medium and 7 in complete medium.

Encapsulated tissue microstructures were also challenged with fulvestrant (ICI 182,780), an ER antagonist and degrader [20–22]. For these experiments, 3–5 days after encapsulation HMEC medium was supplemented with 1 μM fulvestrant (Tocris Bioscience). Twice a week, half volume of culture medium was changed and fulvestrant was replenished to keep a constant concentration. After 2 weeks, samples were centrifuged at 300x g, 5 min at 4 °C, washed with PBS and processed for IHC (as detailed above) or RT-qPCR analysis. Samples for RT-qPCR were stored in RNAlater Stabilization Solution (Roche), according with the manufacturer’s instructions, until further analysis; samples for western blot were snap frozen at − 80 °C. Challenge with fulvestrant was performed in encapsulated microstructures derived from 8 different patients, of which 7 were evaluated by RT-qPCR and 3 by Western Blot.

### Gene expression analysis

Tissue microstructures were thawed, total RNA was extracted in a tissue lyser (Precellys Evolution Homogenizer, Bertin Instruments) and purified using the RNAeasy Kit (Qiagen), according to the manufacturer’s instructions. Reverse transcription was performed using Sensiscript RT kit (Qiagen), also according with the manufacturer’s instructions. qPCR was performed in triplicates, using the SYBR green I Master kit (Roche), in a LightCycler 480 II (Roche). We evaluated expression of ERα (*ESR1)* and its downstream target genes, *pS2*, *AREG* and *PGR* [23], and of two housekeeping genes, *RPL22* [12] and *36B4* [23]. Primer sequences are provided in Table S2. Due to the scarcity of ERα negative BC samples, a ERα and PR negative BC cell line, MDA-MB-231, was employed as basal expressing control [24]. Results are shown as fold change in mRNA amount compared to the vehicle control (CTRL), calculated according to the 2^-ΔΔCt^ method [25], considering a geometric mean of the 2 housekeeping genes used.

### Western blot analysis

Samples were thawed, resuspended in Laemmli Buffer (20% Glycerol, 4% SDS in 100 mM Tris Buffer, pH 6.8) and lysed in a Tissue homogenizer (Precellys Evolution, Bertin Instruments). BC Microstructure lysates were recovered, sedimented to remove cell debris, sonicated and stored at − 80 °C until use.

Protein quantification was performed in a Nanodrop ND-2000C (Thermo Scientific). Proteins were denatured and loaded in a electrophoresis gel (NuPAGE 4–12% Bis-Tris Gel) under reducing conditions for 50 min (200 V) and then electrophoretically transferred using a wet transfer system (Bio-Rad, 30 V, 18 h, 4 °C) into nitrocellulose membranes. Membranes were blocked for 1 h in TBS with 0.1% (w/v) Tween 20, 5% (w/v) non-fat dried milk and further incubated with the primary antibodies (Mouse anti-Human ERα, 1D5 Clone, Dako, final dilution 1:500; Rabbit anti-β tubulin, H-235, SC-9104, SantaCruz, final dilution 1:1000, used as loading control) and respective secondary HRP-conjugated secondary antibodies (Sheep anti Mouse IgG NA931; Donkey anti Rabbit IgG NA934; GE Healthcare, final dilution 1:20000). Membranes were developed using Amersham ECL Select Western Blot Detection Reagent (GE Healthcare) and visualized using a ChemiDoc System (BioRad).

### Statistical analysis

Statistical analysis was performed using GraphPad Prism version 6.0 (GraphPad Software). Data were analyzed as indicated in the figure legends. The Mann-Whitney test was performed to evaluate statistical difference between conditions. Data are presented as mean ± SD, unless otherwise specified.

## Results

### Alginate encapsulated tissue microstructures maintain parental tumor tissue characteristics for at least one month of culture

To establish an ER+ BC ex vivo model, we investigated the possibility of retaining the TME and consequently ERα signaling of patient-derived tissue microstructures immobilized within alginate capsules and cultured under agitation (Fig. 1a). Encapsulated tissue microstructures were cultured for up to 30 days, showing high cell viability, as indicated by maintenance of resazurin reduction capacity along culture time (97 ± 28% by the end of week 4, relatively to the beginning of the culture, Figure S2a). Moreover, detection of extracellular lactate in culture medium (data not shown), as an indicator of high metabolic activity [26], corroborated the high cell viability within the encapsulated tissue microstructures.

**Fig. 2:**
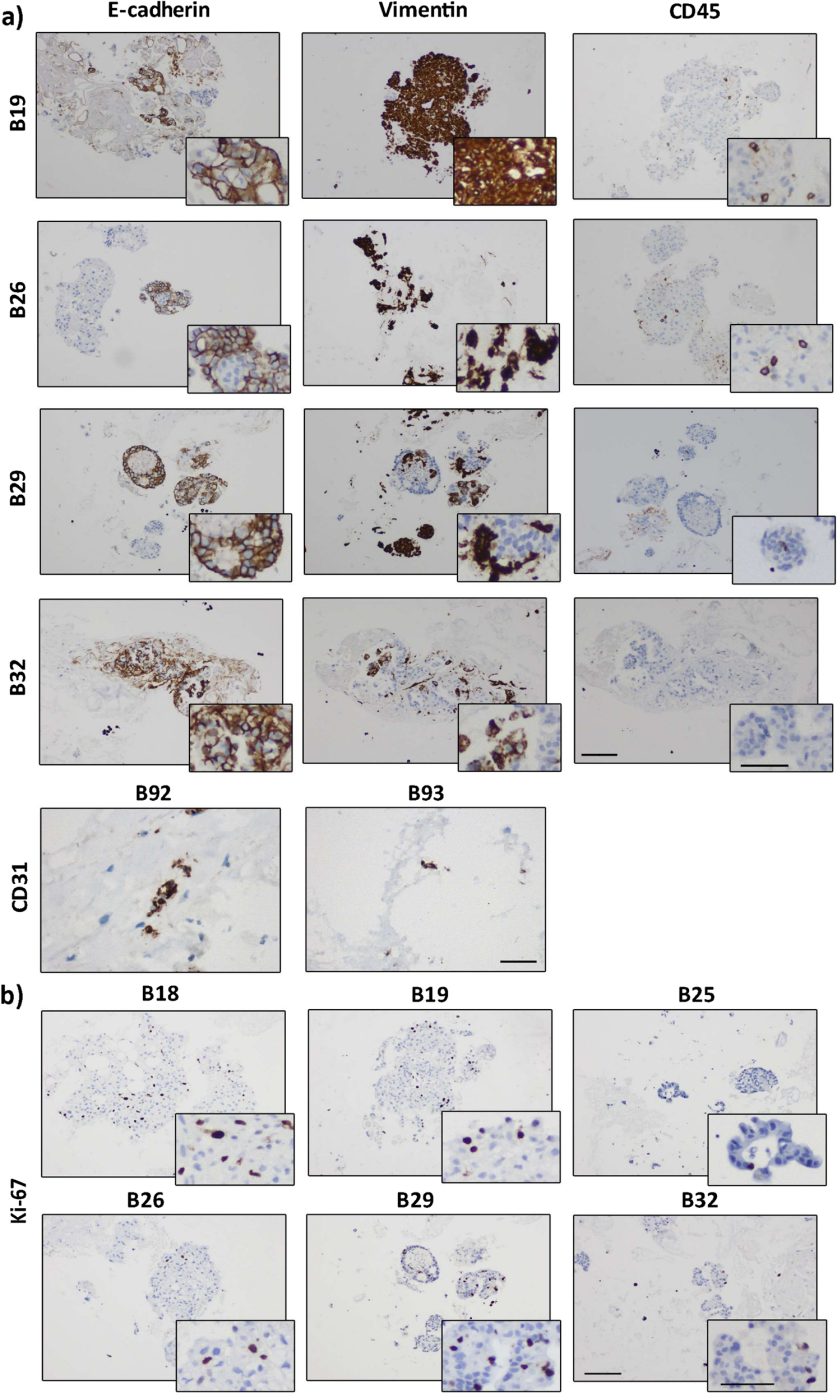
Alginate encapsulated tissue microstructures maintained cell populations and contained proliferating cells. **a** Immunohistochemistry analysis of: E-cadherin (epithelial cells); vimentin (stromal cells); CD45 (immune cells); CD31 (endothelial cells) at 1 month of culture **b** Immunohistochemistry analysis of Ki-67 (cell proliferation) of encapsulated microstructures at 1 month of culture (scale bars: 200 μm for low magnification and 100 μm for high magnification)

**Fig. 3:**
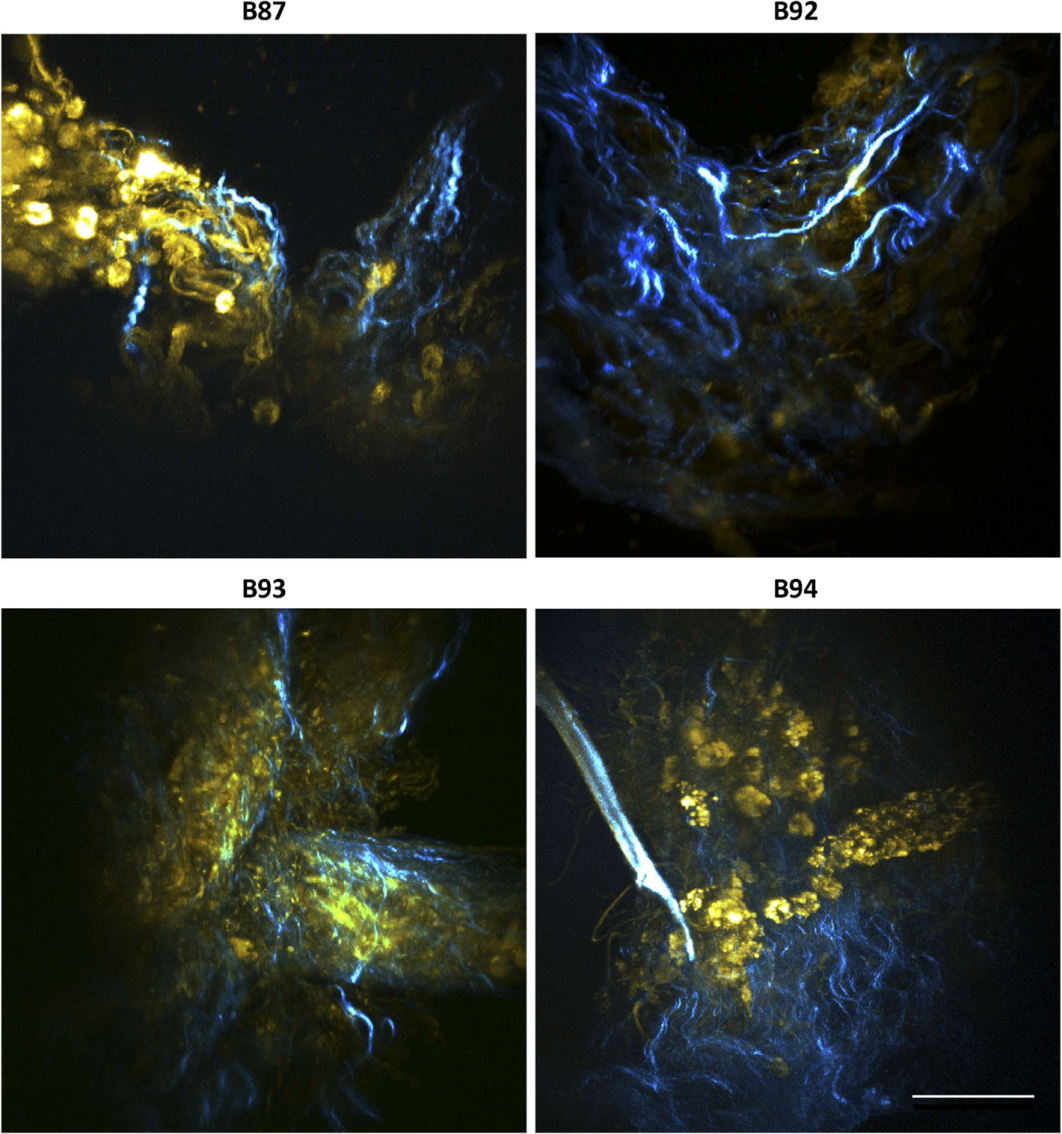
Encapsulated tissue microstructures maintained collagen fibrillar structures Second Harmonic Generation (SHG) microscopy at 1 month of culture: yellow – Two-Photon Excitation Microscopy (TPEF); blue - collagen fibers (scale bar: 50 μm)

The original tumors were very heterogeneous, not only between but also within patients (Fig. 1b): tissue architecture varied in epithelial versus stromal content, cell organization and on the presence/absence of immune cells (CD45+ cells). A complete mixture of malignant epithelial cells and stromal cells was rarely observed. Instead, there were islets of tumor cells surrounded by multiple stromal cells (Fig. 1b, upper panels). These histopathological characteristics were maintained in encapsulated tissue microstructures cultured for a month (Fig. 1b, lower panels). By day 30 of culture, E-cadherin, vimentin, CD31 and CD45 were immunohistochemically-detected (Fig. 2a). The detection of membranous E-cadherin indicated that carcinoma cells maintained the typical cell-cell adhesions and differentiated phenotype [27]. On the other hand, vimentin detection confirmed the presence of stromal cells. CD45, also known as leucocyte common antigen, is a transmembrane glycoprotein present in all nucleated cells of the hematopoietic lineage [28] and has been broadly used to assess immune cell population presence in breast tissue, such as tumor-infiltrating lymphocytes [29–31]. CD45^+^ cells were detected in 5 out of the 8 cases which presented immune cells in the original tissue (Fig. 2a). In two analyzed tissue microstructures, CD31 positivity confirmed the presence of endothelial cells (Fig. 2a). Absence of cells positive for the basal/myoepithelial marker p63 was observed similarly to the original tumors (Figure S2b). Ki67-positive cells were also detected at different levels, indicating the presence of proliferating cells even after 1 month of culture (Fig. 2b). Although at low levels, this is consistent with the parental tissues, where the median of proliferating cells was 20% (Q1 = 15; Q3 = 30). Second harmonic generation analysis (SHG) of encapsulated BC tissue microstructures revealed dense and organized/fibrillar collagen fibers in peripheral regions of the samples analyzed, surrounding areas of cellularity (Fig. 3). As a culture control, non-encapsulated tissue microstructures were cultured in parallel. A significant decrease in resazurin reduction ability after 3–4 weeks of culture was observed, suggesting a reduced cell viability of these cultures. Remarkably, cell viability was increased in encapsulated versus non-encapsulated tissue microstructures (Figure S2c).

Altogether, we were able to extend the lifespan of BC explant cultures for up to 1 month whilst maintaining tissue architecture, the different cell types of the BC microenvironment, and cell viability.

### ERα expression and functionality are sustained over 1 month of culture

**Fig. 4:**
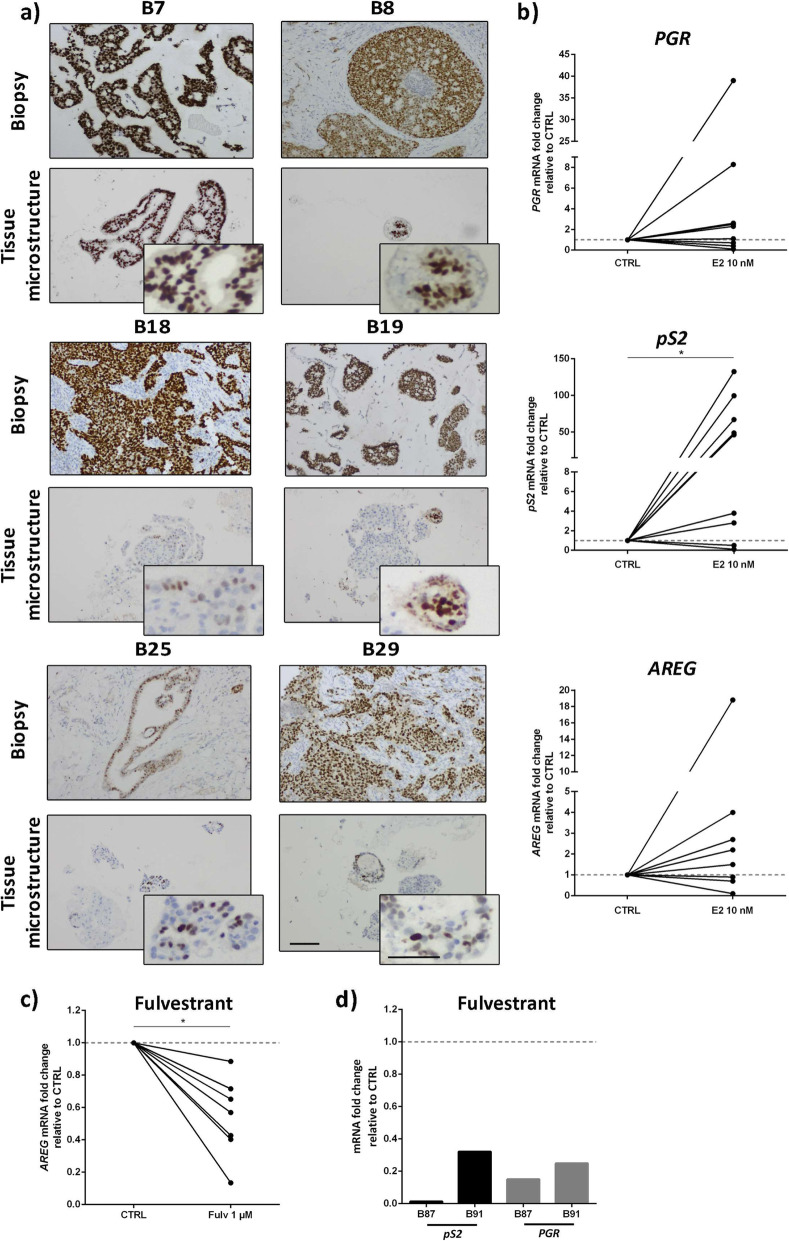
Estrogen Receptor α (ER) expression and functionality were maintained in alginate encapsulated tissue microstructures up to 1 month of culture. **a** Immunohistochemistry detection of ER in biopsy (top row) and encapsulated tissue microstructures culture for a month (bottom row) (scale bars: 200 μm for low magnification and 100 μm for high magnification). **b** Encapsulated tissue microstructures were cultured for 3 days in depleted medium and stimulated with 17-β-estradiol; expression of ER downstream target genes was assessed by RT-qPCR (amphiregulin - *AREG*, progesterone receptor - *PGR* and protein PS2 - *pS2*, *N* = 9). Data are shown as fold change in gene expression upon 17-β-estradiol challenge relatively to vehicle-exposed control (CTRL). **c**, **d** Encapsulated tissue microstructures were cultured for 3–5 days in complete medium, before challenge with fulvestrant for 2 weeks; ER downstream targets were assessed by RT-qPCR (*AREG, PGR* and *pS2*, *N* = 7). Data are shown as fold change in gene expression upon fulvestrant challenge relatively to vehicle-exposed control. Statistical analysis was performed by the Mann-Whitney test (*p-value < 0.001)

After 1 month in culture, ER+ carcinoma cells were still detected in the encapsulated tissue microstructures by IHC analysis (Fig. 4a), typically in lesser extent that in the original sample. When sample material was not sufficient for IHC evaluation, mRNA was quantified, relatively to MDA-MB-231, a human cancer cell line which does not express ERα nor PR [24] (Figure S3a). All samples presented higher expression of the ERα gene (*ESR1)* than MDA-MB-231 cells, indicating ERα gene expression after 1 month of culture (Figure S3b and Figure S5).

To assess ERα function, encapsulated tissue microstructures derived from ER+ BC from 9 distinct patients were stimulated with 10 nM 17-β-estradiol for 24 h and the mRNA levels of the ER target genes evaluated: protein PS2 (also known as Trefoil Factor 1 -TFF1-, *pS2)*, progesterone receptor (*PGR*) and amphiregulin (*AREG*) [23]. *AREG* and *PGR* were upregulated upon challenging with 17-β-estradiol compared to vehicle-controls (mean fold increase in *AREG* and *PGR* expression of 3.4 ± 5.6 and 6.3 ± 11 respectively, Fig. 4b, Figure S4a). Strikingly, we detected a generalized upregulation of *pS2* (in 7 out of 9 tissue microstructures), with a mean fold increase in gene expression of 45 ± 45, compared to the vehicle-treated control (Fig. 4b, Figure S4a and Figure S5). In general, there was a trend for a positive correlation between *ESR1* basal expression and the expression of the three ER target genes upon estrogen challenge (Figure S4b, *R* > 0 by Pearson Correlation), even though not significant, probably due to the intrinsic variability of primary tumors and the sample size. Tissue microstructure cultures derived from ER-negative BC tumors were also treated with 17-β-estradiol and no upregulation of ER downstream genes was observed (Figure S4c). This data further corroborates that the original phenotype was maintained in culture. A different set of encapsulated tissue microstructures, derived from 7 ER+ BC samples, were maintained in HMEC medium until 17- β-estradiol challenge. These showed a mild stimulation of ERα target genes (on average, 2.3-, 1.8- and 1.2-fold increase relatively to vehicle control for *pS2*, *PGR* and *AREG*, respectively, Figure S4d).

To further confirm intact ERα signaling in encapsulated tissue microstructures, cultures derived from additional ER+ BC samples were exposed to fulvestrant (or ICI 182,780), a ERα full antagonist [32] widely used in endocrine therapy [33]. After 2 weeks of exposure, a generalized down regulation of *AREG* compared to vehicle controls was observed (Fig. 4c). For two of the tumors, we also evaluated *PGR* and *pS2* response and observed a strong reduction of mRNA levels compared to vehicle controls (Fig. 4d). Additionally, we assessed ERα protein levels in three of the tumors and observed a tendency for reduction compared to vehicle control conditions (Figure S6). Collectively, these results indicate that *ERα* is expressed in encapsulated tissue microstructures derived from ER+ BC samples and can respond to stimulation and inhibition.

## Discussion

ERα signaling is considered a defining and driving event contributing to ER+ BC carcinogenesis; ERα overexpression in primary tumors has been linked to disease progression, influencing patient survival [34–36]. Nonetheless, approximately 30% of patients with ER+ BC fail to respond to endocrine therapy [2]. Several reports have shown the intricate relation between response to therapy and TME components, such as fibroblasts [37–39] and ECM components [40, 41]. Therefore, it is paramount to define the biological determinants of ERα intra-tumoral heterogeneity and the mechanisms underlying therapeutic resistance. However, this knowledge has been hampered by the challenges in developing experimental models recapitulative of intra-tumoral ERα heterogeneity and in which ERα signaling is sustained, essential to address long-term effects of tumor-stromal interactions in ERα signaling and drug response mechanisms against ER.

Here, we propose a culture strategy in which patient-derived tissue microstructures retain ER+ carcinoma cells for at least 1 month of culture; of note, these cells still respond to ER stimulation and inhibition, therefore constituting a functional ex vivo model of ER-positive BC. Tissue microstructures, that were entrapped in alginate capsules and cultured under dynamic conditions, maintained high cellularity and low levels of tumor cell proliferation, as reported for human ER+ BC [42], and parental tissue architecture (including epithelial, stromal and endothelial cell compartments and deposited fibrillar collagen). Although all interrogation was limited to 1 month of culture, as we have not detected signs of tissue microstructure decline in cell viability up to that timepoint, we conjecture that the lifespan of encapsulated tissue microstructures could be extended for even longer periods.

We hypothesized that using tissue microstructures within the millimeter size range would be more favorable to attain an accurate representation of intra-tumoral heterogeneity and TME, than more miniaturized ex vivo models. To overcome the major limitations of ex vivo cultures – the reduced lifespan and zonation due to diffusional gradients [43], we resourced to dynamic culture conditions. Agitation improves mass transfer, promoting nutrient and oxygen diffusion, reducing the formation of gradients typically observed for tissue microstructures within the above mentioned size range [44, 45]. Moreover, we encapsulated in alginate, a biocompatible, inert hydrogel [46] since it has defined composition and confers support and protection from agitation-induced shear stress [13, 47, 48]. This contributes to the preservation of tissue architecture and cell viability, but also promotes the built-up of relevant cell microenvironment factors. In fact, we have previously shown that cells entrapped in alginate capsules, and cultured under agitation, accumulate secreted soluble factors (e.g., cytokines) and ECM components, promoting homotypic and heterotypic cellular crosstalk, cell migration and reconstruction of cancer-related microenvironments [12, 13], such as an immunosuppressive microenvironment in a non-small cell lung cancer model [12]. In terms of ECM components, we not only observed the maintenance of TME cellular components in the encapsulated tissue microstructures, such as the stromal cells, which are involved in the secretion of collagen [49], but also ECM components as collagen fibers. These were detected by SHG microscopy, a technique broadly applied to BC tissue [50]. In all the encapsulated tissue microstructures analyzed fibrillar collagen presence was observed. Increased collagen density has been shown to directly promote BC tumorigenesis [51]. Moreover, collagen is strongly associated with mammographic density used as a measurement of risk of BC [52] and is responsible for drug resistance since it prevents the penetration of therapeutic agents, such as antibodies [53].

The preservation of tumor heterogeneity and TME are critical to closely mimic the in vivo situation [4, 54]. We observed a high degree of heterogeneity between distinct parental tissues - not only the levels of ER-positivity were different, but also the percentages and physical distribution of carcinoma and stromal cells - that were recapitulated in the derived tissue microstructures. In 5 out of 8 tissue microstructure cultures derived from tumors with immune cell infiltrate, CD45-positive cells were retained even after 1 month of culture, although in low amounts. This is in accordance with the typically low frequency of immune cell infiltrates in ER+ tumors [55].

After 1 month in culture, p63 was not detected in tissue microstructures, in accordance with what is reported for luminal BC. The myoepithelial marker p63 is present in basal cells of a variety of healthy epithelial tissues [56], such as in normal breast tissue. However, its expression in BC is rare [56, 57]. On the other hand, tissue microstructures presented low levels of Ki-67; in fact, ERα-positive subtypes have lower proliferative indexes than other BC subtypes [58]. The intrinsic low levels of cell proliferation and the reduced amount of patient tissue available to set-up tumor microstructure cultures, limit their application in high throughput assays.

The maintenance of ER+ cells in culture is a major accomplishment, as ERα ablation ex vivo has been a major issue in ER+ BC research [59]. The sustained expression of ERα is pivotal for the study of the luminal A subtype of BC, as cell proliferation is ER-dependent and targeted therapies usually rely on prolonged treatment with ERα antagonists [60]. After 1 month in culture, we detected ER+ cells in the encapsulated tissue microstructures, typically in a less extent than in the original tumor. ERα functionality was evaluated by challenging encapsulated tissue microstructures, with either activator (17-β-estradiol) or inhibitor (fulvestrant) molecules. Our results show differential expression of *PGR*, *AREG* and *pS2* in tissue microstructures originated from different ER+ BC patients, suggesting that the model reflects inter-patient heterogeneity. This may be in terms of basal expression levels of the target genes analyzed, ER transcriptional response and potential presence of ER-independent regulatory pathways [4, 61]. *pS2* is a well-known direct downstream ERα target, which is under the positive control of an ERE consensus sequence located 400 bp before transcription starting site [62]. Our results show a higher upregulation of *pS2* when comparing with *AREG* and *PGR*. In fact, it has been reported for the ER+ MCF-7 BC cell line that, upon estrogen exposure, *pS2* expression strongly increases compared to *PGR*, at mRNA and also at protein levels [63, 64]. We have also observed the effects of fulvestrant at the level of ERα protein, as the drug is described to accelerate ERα degradation [32].

Aiming to retain ER+ cells, we employed a culture medium enriched in molecules with reported ER stimulatory effects, such as insulin, hydrocortisone and EGF [15–20]. 17-β-estradiol and EGF may also be produced by the breast fibroblasts present in culture [65–67]. Our observation of reduced effects upon 17-β-estradiol stimulation in tissue microstructures cultured in complete medium compared with tissue microstructures cultured in depleted medium in the 3 days preceding stimulation, corroborates the presence of soluble ER activators in culture. Further studies are required to understand the signaling events that contribute to the maintenance of ERα signaling under the culture conditions here presented, which will potentially also contribute to further disclose its role in ER+ BC.

## Conclusions

Overall, we advocate a new methodology for ER+ BC TME modelling, in which the original cell populations, the native ECM and tissue architecture are represented, and ER function sustained. This ex vivo culture system can contribute to the study of breast cancer biology, in particular ERα signaling and microenvironmental-driven molecular mechanisms. Moreover, due to the extended culture time, the system can be a useful tool to study novel anti-endocrine therapies and other therapeutic modalities.

## Supplementary information

**Additional file 1: Figure S1.** Sample weight.

**Additional file 2: Figure S2.** **a** Metabolic activity was assessed along culture. **b** Immunohistochemistry analysis of p63 (myoepithelial cells) at 1 month of culture (scale bar: 60 μm). **c** Metabolic activity was assessed in encapsulated and non-encapsulated tissue microstructures derived from the same patients.

**Additional file 3: Figure S3. a** Hematoxylin and eosin staining and immunohistochemistry for ERα of MDA-MB-231 (ER-negative cell line) cells cultured in 2D (scale: 200 μm). **b** ERα gene (*ESR1)* expression in encapsulated microstructures cultured for 1 month relatively to MDA-MB-231 cells.

**Additional file 4: Figure S4. a** Encapsulated tissue microstructures were cultured for 3 days in depleted medium before stimulation with 17-β-estradiol; expression of ER downstream target genes was assessed by RT-qPCR (amphiregulin - *AREG*, progesterone receptor *- PGR* and protein PS2 - *pS2*, *N* = 9); quantitative evaluation of data shown in Fig. 3b). **b** Correlation diagrams of expression of ERα gene (*ESR1*) and ER target genes (*PGR, pS2* and *AREG*). The dots represent the log (mRNA fold change relative to control) of each gene for a given BC patient microtissue and the lines represent the linear regression (Pearson correlation with R indicated in each graph). For all cases, no significant correlation was found (*p-value* > 0.7).**c** ER-negative BC encapsulated tissue microstructures cultured in complete medium were challenged with 17-β-estradiol and expression of ER downstream target genes was assessed by RT-qPCR (*AREG*, *PGR pS2*, *N* = 2). **d** Encapsulated tissue microstructures cultured in complete medium were challenged with 17-β-estradiol and ER downstream target genes were assessed by RT-qPCR (*AREG*, *PGR* and *pS2, N* = 7). Data are shown as fold-change in gene expression upon 17-β-estradiol challenge relatively to vehicle-exposed control (CTRL).

**Additional file 5: Figure S5.** Encapsulated tissue microstructures were cultured for 3 days in depleted medium before stimulation with 17-β-estradiol; expression of ER downstream target genes was assessed by RT-qPCR (amphiregulin - *AREG*, progesterone receptor *- PGR* and protein PS2 - *pS2*). Data is presented individually for each tumor.

**Additional file 6: Figure S6.** Encapsulated tissue microstructures were cultured for 3–5 days in complete medium, before challenge with fulvestrant for 2 weeks; ERα protein was detected by western blot; β-tubulin was used as loading control (*N* = 3, representative blot out of 2 technical replicates).

**Additional file 7: Table S1.** Immunohistochemistry analysis: reagents and conditions.

**Additional file 8: Table S2.** RT-qPCR analysis: primer sequences.

## Data Availability

All data generated or analyzed during this study are included in this published article (and its supplementary information files).

## Abbreviations

AREG: Amphiregulin
BC: Breast cancer
CTRL: Control
ERα: Estrogen receptor α
ER+ BC: Estrogen receptor α-positive breast cancer
H&E: Hematoxylin and eosin
IHC: Immunohistochemistry
PRG: Progesterone
SHG: Second harmonic generation
TME: Tumor microenvironment
TPEF: Two-photon-excited fluorescence
