# Supplementary figures and images for: A novel culture method that sustains ERα signaling in human breast cancer tissue microstructures
Source: J Exp Clin Cancer Res. 2020 Aug 17;39:161. doi: 10.1186/s13046-020-01653-4 (PMC7430012; doi:10.1186/s13046-020-01653-4)

Figure S1

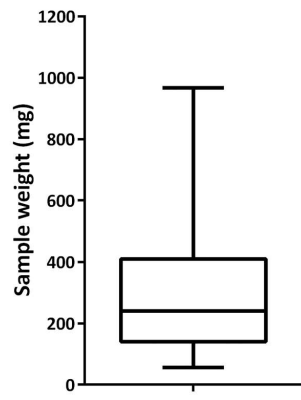

Supplement: Supplementary file 1 — Additional file 1: Figure S1. Sample weight. [file 13046_2020_1653_MOESM1_ESM.pdf]

**Figure S2**

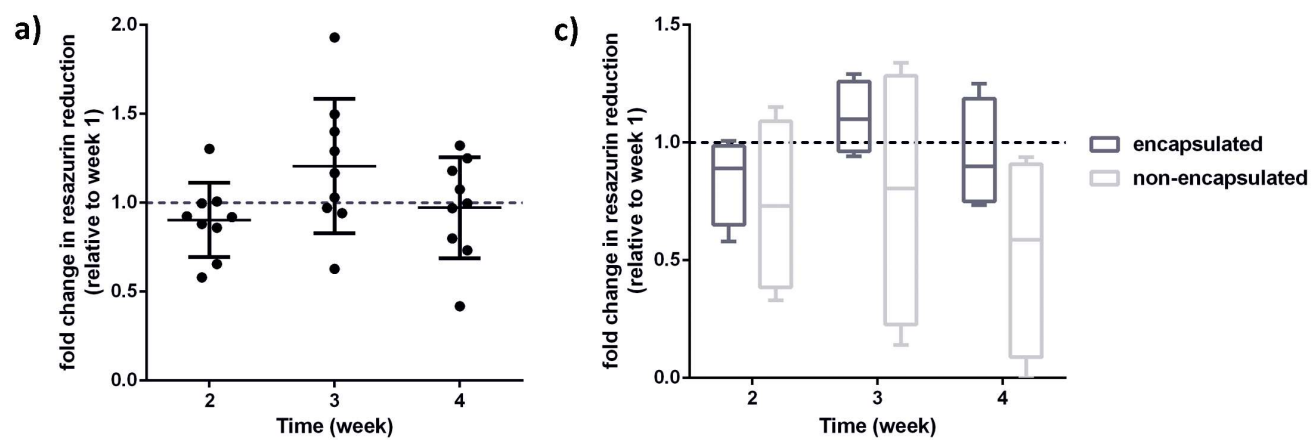

**b)**

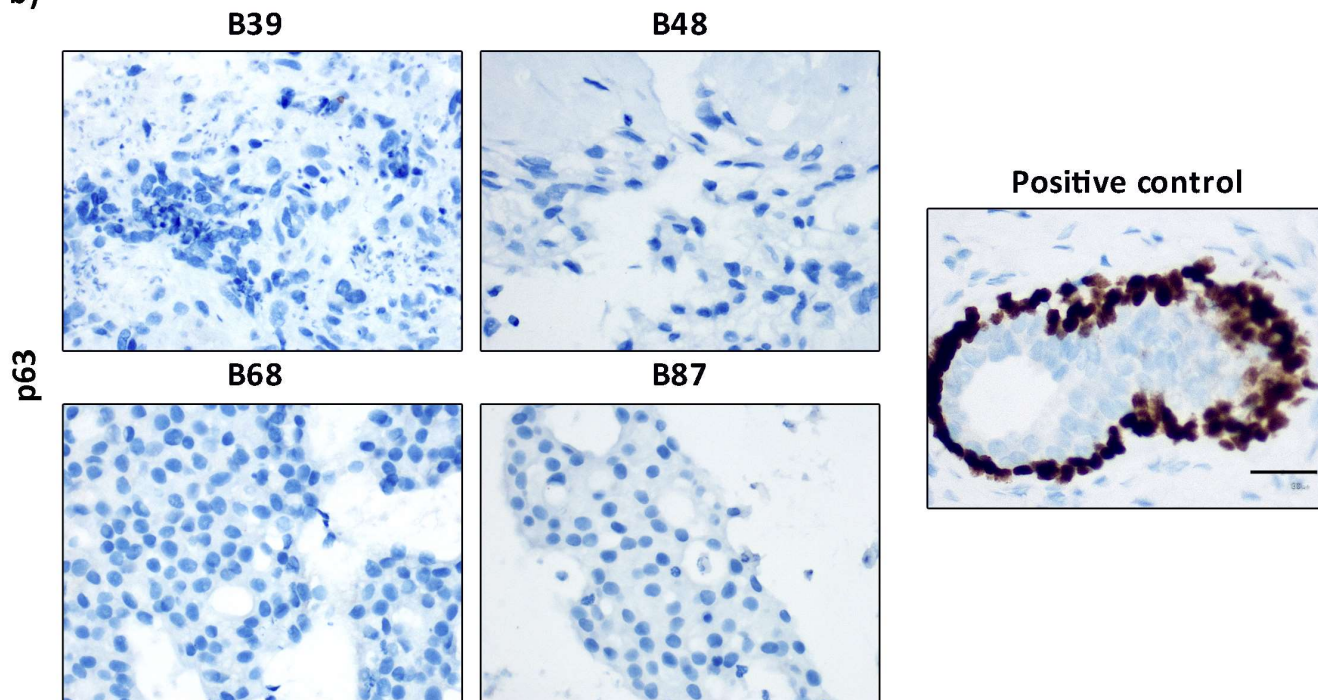

Supplement: Supplementary file 2 — Additional file 2: Figure S2. a Metabolic activity was assessed along culture. b Immunohistochemistry analysis of p63 (myoepithelial cells) at 1 month of culture (scale bar: 60 μm). c Metabolic activity was assessed in encapsulated and non-encapsulated tissue microstructures derived from the same patients. [file 13046_2020_1653_MOESM2_ESM.pdf]

Figure S3

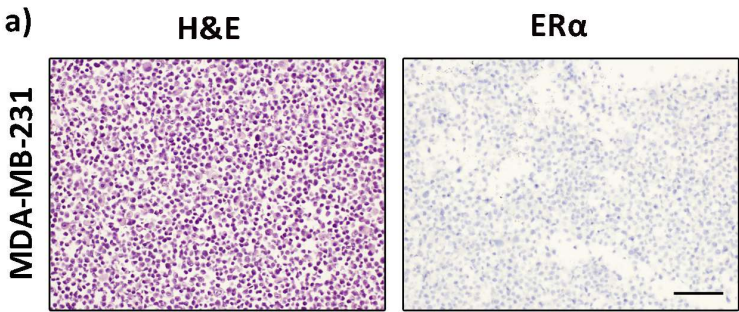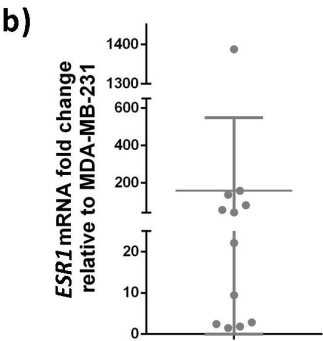

Supplement: Supplementary file 3 — Additional file 3: Figure S3. a Hematoxylin and eosin staining and immunohistochemistry for ERα of MDA-MB-231 (ER-negative cell line) cells cultured in 2D (scale: 200 μm). b ERα gene (ESR1) expression in encapsulated microstructures cultured for 1 month relatively to MDA-MB-231 cells. [file 13046_2020_1653_MOESM3_ESM.pdf]

**Figure S4**

**a)**

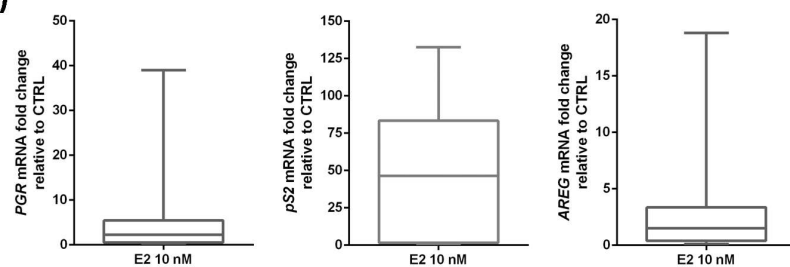

**b)**

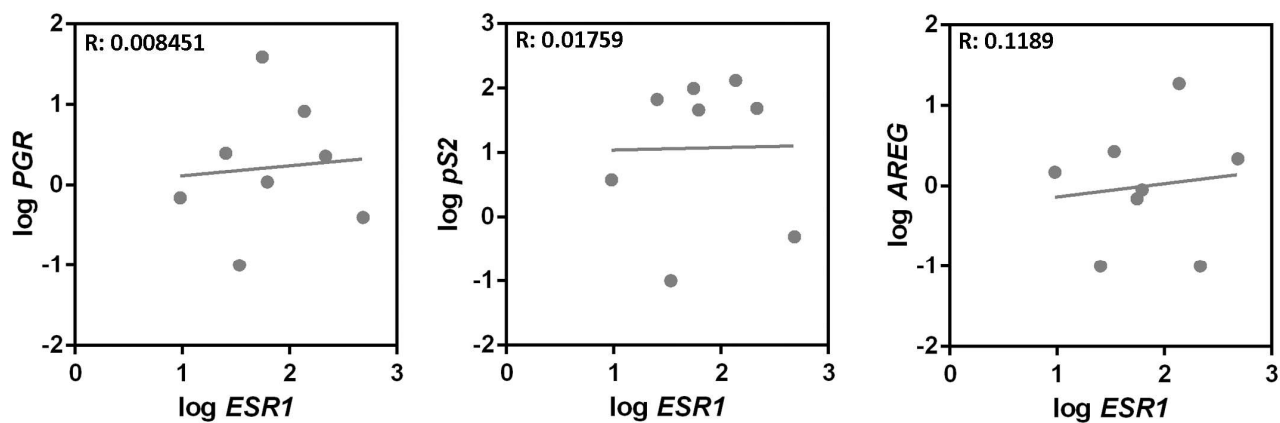

**c)**

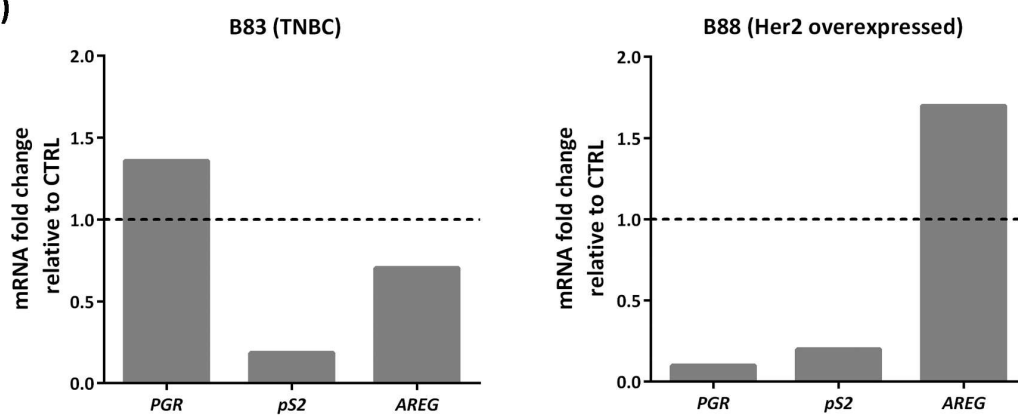

**d)**

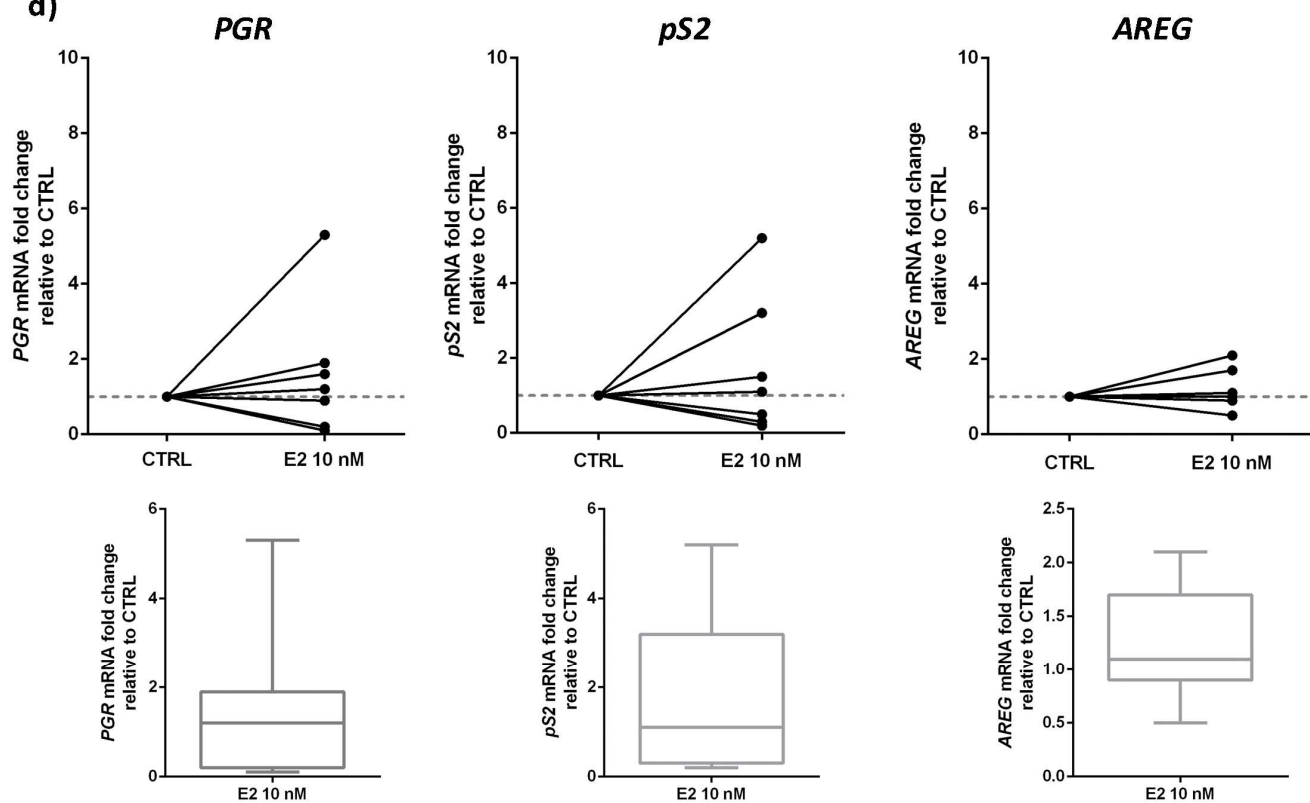

Supplement: Supplementary file 4 — Additional file 4: Figure S4. a Encapsulated tissue microstructures were cultured for 3 days in depleted medium before stimulation with 17-β-estradiol; expression of ER downstream target genes was assessed by RT-qPCR (amphiregulin - AREG, progesterone receptor - PGR and protein PS2 - pS2, N = 9); quantitative evaluation of data shown in Fig. 3b). b Correlation diagrams of expression of ERα gene (ESR1) and ER target genes (PGR, pS2 and AREG). The dots represent the log (mRNA fold change relative to control) of each gene for a given BC patient microtissue and the lines represent the linear regression (Pearson correlation with R indicated in each graph). For all cases, no significant correlation was found (p-value > 0.7).c ER-negative BC encapsulated tissue microstructures cultured in complete medium were challenged with 17-β-estradiol and expression of ER downstream target genes was assessed by RT-qPCR (AREG, PGR pS2, N = 2). d Encapsulated tissue microstructures cultured in complete medium were challenged with 17-β-estradiol and ER downstream target genes were assessed by RT-qPCR (AREG, PGR and pS2, N = 7). Data are shown as fold-change in gene expression upon 17-β-estradiol challenge relatively to vehicle-exposed control (CTRL). [file 13046_2020_1653_MOESM4_ESM.pdf]

**Figure S5**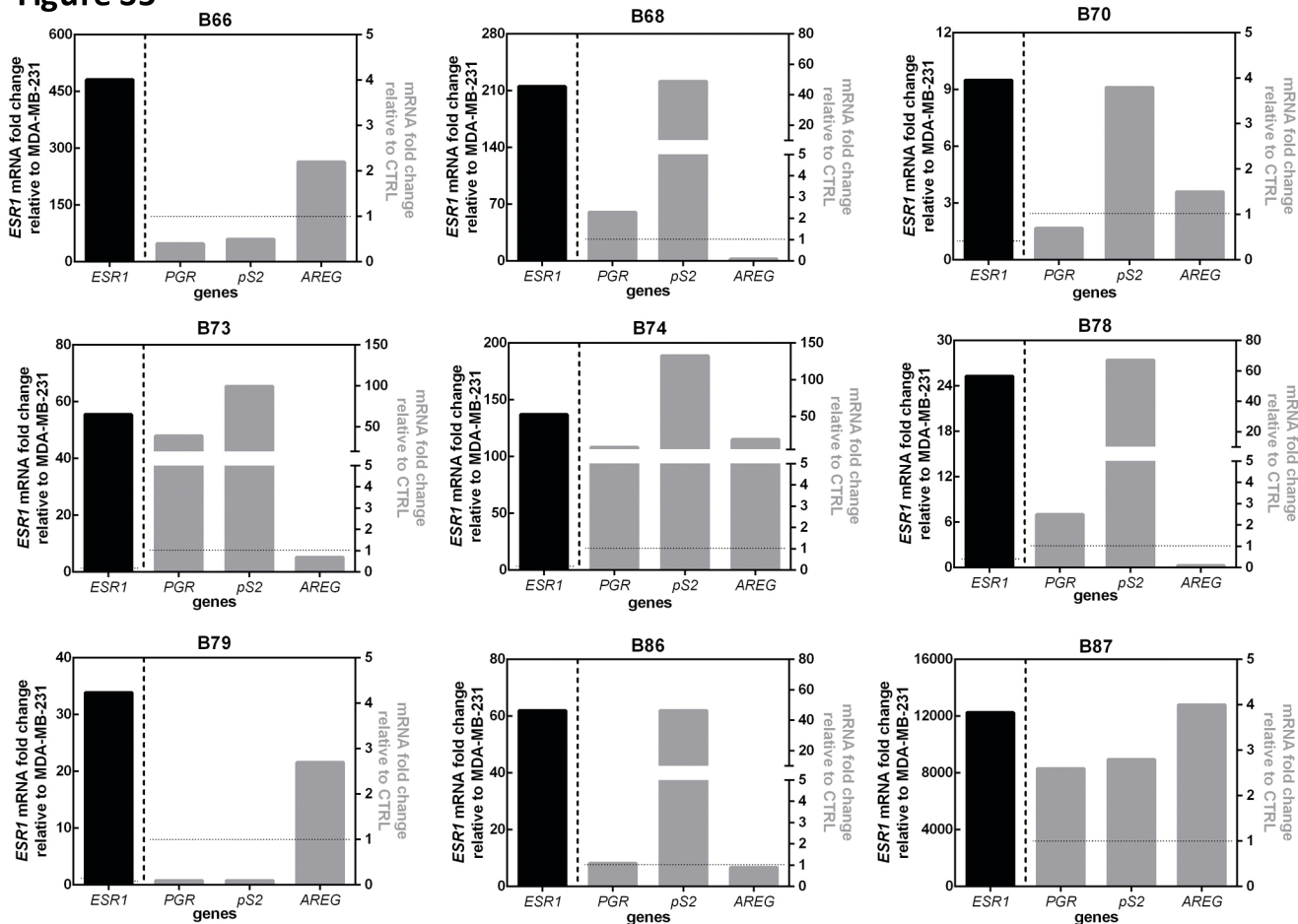

Supplement: Supplementary file 5 — Additional file 5: Figure S5. Encapsulated tissue microstructures were cultured for 3 days in depleted medium before stimulation with 17-β-estradiol; expression of ER downstream target genes was assessed by RT-qPCR (amphiregulin - AREG, progesterone receptor - PGR and protein PS2 - pS2). Data is presented individually for each tumor. [file 13046_2020_1653_MOESM5_ESM.pdf]

Figure S6

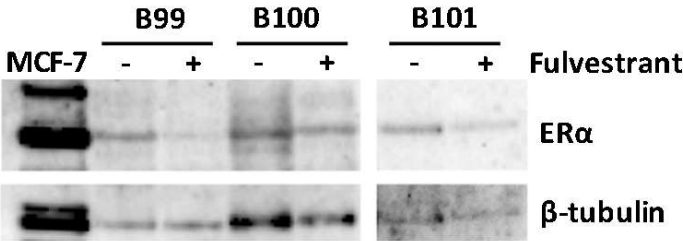

Supplement: Supplementary file 6 — Additional file 6: Figure S6. Encapsulated tissue microstructures were cultured for 3–5 days in complete medium, before challenge with fulvestrant for 2 weeks; ERα protein was detected by western blot; β-tubulin was used as loading control (N = 3, representative blot out of 2 technical replicates). [file 13046_2020_1653_MOESM6_ESM.pdf]
