# Supplementary material for: A novel culture method that sustains ERα signaling in human breast cancer tissue microstructures
Source: J Exp Clin Cancer Res. 2020 Aug 17;39:161. doi: 10.1186/s13046-020-01653-4 (PMC7430012; doi:10.1186/s13046-020-01653-4)
Supplement: Supplementary file 7 — Additional file 7: Table S1. Immunohistochemistry analysis: reagents and conditions. [file 13046_2020_1653_MOESM7_ESM.docx]

**Table S1:** Immunohistochemistry analysis: reagents and conditions used for.

| Primary antibody clone | Supplier | Dilution | Antigen retrieval | Detection system |
| --- | --- | --- | --- | --- |
| E-cadherin (NCH 38) | Dako  (M3612) | 1:80  (28 minutes) | 40 min with CC1* | Optiview# |
| Oestrogen receptor (SP1) | Ventana  (28 minutes) | Pre-diluted  (28 minutes) | 64 min with CC1* | Ultraview# |
| KI67 (30-9) | Ventana  (790-4286) | Pre-diluted  (16 minutes) | 32 min with CC1* | Optiview# |
| CD45 (2B11-PD7/26) | Dako  (M0701) | 1:1000  (32 minutes) | 56 min with CC1* | Optiview# |
| Vimentin (clone V9) | Dako  (M0725) | 1:150  (12 minutes) | 24 min with CC1* | Optiview# |
| P63 (clone 4A4) | Biocare Medical  (CM163C) | 1:200  (24 minutes) | 48 min with CC1* | Optiview# |
| CD31 (clone 1A10) | Novocastra  (NCL-CD31-1A10) | 1:80  (16 minutes) | 32 min with CC1* | Optiview# |
