# Supplementary material for: A novel culture method that sustains ERα signaling in human breast cancer tissue microstructures
Source: J Exp Clin Cancer Res. 2020 Aug 17;39:161. doi: 10.1186/s13046-020-01653-4 (PMC7430012; doi:10.1186/s13046-020-01653-4)
Supplement: Supplementary file 8 — Additional file 8: Table S2. RT-qPCR analysis: primer sequences. [file 13046_2020_1653_MOESM8_ESM.docx]

**Table S2:** RT-qPCR analysis: primer sequences.

| Symbol | Gene name | Forward Primer (5'-3') | Reverse primer (3'-5') |
| --- | --- | --- | --- |
| *ESR1* | Estrogen receptor α | CCACCAACCAGTGCACCATT | GGTCTTTTCGTATCCCACCTTTC |
| *PGR* | Progesterone receptor | CGCGCTCTACCCTGCACTC | TGAATCCGGCCTCAGGTAGTT |
| *pS2* | Protein PS2/  Trefoil factor 1 | TCGGGGGTCGCCTTTGGAGCAG | GAGGGCGTGACACCAGGAAAACCA |
| *AREG* | Amphiregulin | TGGAAGCAGTAACATGCAAATGTC | GGCTGCTAATGCAATTTTTGATAA |
| *RPL22* | Ribosomal protein L22 | CACGAAGGAGGAGTGACTGG | TGTGGCACACCACTGACATT |
| *36B4* | Acidic ribosomal phosphoprotein P0 | GTGTTCGACAATGGCAGCAT | GACACCCTCCAGGAAGCGA |
